# Patient-reported outcome tools of acupuncture clinical trials in mainland China: a cross-sectional study

**DOI:** 10.3389/fneur.2025.1520759

**Published:** 2025-06-06

**Authors:** Feng Cao, Yue Dong, Zhi-Qiang Li, Xue-Feng Wang, Cheng-Yuan Su, Jia-Xuan Lv, Zhong-Yu Shi, Ming-Hong Du, Xin-Yue Zhang, Hong-Guo Rong, Yu-Tong Fei

**Affiliations:** ^1^Centre for Evidence-Based Chinese Medicine, Beijing University of Chinese Medicine, Beijing, China; ^2^School of Traditional Chinese Medicine, Beijing University of Chinese Medicine, Beijing, China; ^3^Institute of Basic Research in Clinical Medicine, China Academy of Chinese Medical Sciences, Beijing, China; ^4^Institute for Excellence in Evidence-Based Chinese Medicine, Beijing University of Chinese Medicine, Beijing, China

**Keywords:** acupuncture, clinical trials, patient-reported outcome, cross-sectional study, outcome research

## Abstract

**Background and objective:**

Nowadays, the number of acupuncture clinical trials is dramatically increasing. In acupuncture clinical research, patient-reported outcome measurements are important evaluation tools, but there is a lack of systematic survey. This study aims to evaluate the characteristics and application of PRO measurements in acupuncture clinical trials in mainland China, further exploring and developing patient-reported outcomes (PROs) that are in line with the characteristics of acupuncture treatment.

**Methods:**

This cross-sectional study analyzed acupuncture clinical trials in mainland China (2010–2022). Data were extracted from ClinicalTrials.gov and the Chinese Clinical Trial Registry. Acupuncture interventional clinical trials conducted or recruited in mainland China were included. For each included trial, data were extracted on aspects including the clinical trial phase, study setting, participant age, disease, and PRO measurements. Descriptive statistics were performed using Stata 14.0 (StataCorp). Microsoft Excel 2020 (Microsoft Corporation, Redmond, WA, United States) and python3.9 (Netherlands) were used to analyze and display the PROs data.

**Results:**

Out of a total of 962 trials, 193 trials listed PROs as primary outcomes, 208 trials listed PROs as secondary outcomes, and 342 trials listed PROs as co-primary outcomes. Musculoskeletal symptoms (13.5%), neurological disorders (11.7%), and mental health conditions (9.6%) were the most common conditions assessed by PRO tools. The Visual Analogue Scale (VAS) was the most frequently used measurement (30%), followed by concepts related to health-related quality of life (HRQOL). The Pittsburgh Sleep Quality Index (PSQI), 36-Item Short Form Survey (SF-36), and Self-Rating Depression Scale (SDS) were the most common PRO tools utilized in these trials. Clinical trials incorporating PROs were predominantly conducted in the eastern, northern, and southwestern regions of mainland China. Only a part of acupuncture clinical trials (15.2%) used placebos and reported PRO.

**Conclusions and relevance:**

In this cross-sectional study, the use of PROs has increased over the past few decades based on acupuncture clinical trials conducted in mainland China. Given the uneven distribution and lack of acupuncture-specific PROs in the application of acupuncture clinical trials, further attention should be paid to the standardization and regulation of acupuncture-specific scales in the field of acupuncture clinical research.

## 1 Introduction

Patient-reported outcomes (PROs) are documented and collected directly from the patient, without any interpretation of the patient’s response from a clinician or another individual (1, 2). They report on key domains such as symptoms or symptom burden, health-related quality of life, and health behaviors (3). PROs are valuable for shared decision-making and research (4). Patient-reported outcome tools (PRO tools) can collect information from multiple dimensions such as psychological state, physiological function, social activities, diagnostic and treatment satisfaction. From the patient’s perspective, the current health status or quality of life can be assessed (2).

PRO tools are questionnaires that collect health outcomes directly from the people who are suffering, and they are often the best method for measuring patient symptoms and quality of life, helping to reduce clinical researcher bias, and engaging patients in the research process, which can increase the robustness of the study and provide information for health service resource planning, maximizing economic value and improving patient outcomes. There is a range of tools available to assist clinicians and researchers in selecting and using patient-reported outcome tools (5). Many patient-reported outcome tools have been developed and studied in research fields such as musculoskeletal oncology, endocrinology, and cancer to assess the functional outcomes and health-related quality of life of these patients (6–8). Additionally, patient-reported outcome tools are applied to support clinical decision-making, prioritize patients for surgical procedures, compare outcomes among healthcare providers, stimulate quality improvement, and evaluate practices and policies (9).

Traditional Chinese Medicine (TCM) has a history of thousands of years and is an important part of the world’s traditional medical science. As an important component of TCM, acupuncture has attracted more and more attention worldwide for its unique therapeutic effects. Acupuncture treatment, which consists of acupuncture dosage, diagnosis and communication between doctors and patients during the treatment process, the professional knowledge of the acupuncturist, and the individualization of the intervention, is a complex intervention. It is a key issue to conduct an objective, scientific, and systematic evaluation of the clinical efficacy of acupuncture (10).

In the clinical diagnosis and treatment process of TCM and acupuncture, great emphasis is placed on the holistic and dialectical view, focusing on the patient’s subjective feelings and symptom improvement. This characteristic aligns with the new medical model of “bio-psycho-social” that emphasizes a multi-dimensional perspective on health and disease from the patient’s social function, psychological and mental state, self-satisfaction, and sense of wellbeing. This provides a theoretical basis for introducing the PRO evaluation model into the evaluation of the clinical efficacy of acupunctures (11).

PRO tools play a crucial role in acupuncture clinical research, providing effective information about the individual experiences and treatment responses of patients, which helps to better evaluate the efficacy of acupuncture and supports patient-centered clinical decision-making (12). In recent years, the number of acupuncture clinical trials conducted in mainland China has shown continuously increasing trends (13). Whether acupuncture treatment has a placebo effect has always been a hot topic in clinical research (14). Therefore, it is of great significance to conduct a comprehensive survey of the use of PROs in acupuncture randomized controlled trials, which can provide reference suggestions for conducting high-quality acupuncture clinical trials. This study is based on the registration information of acupuncture randomized clinical trials conducted domestically, aimng to review and evaluate the use of PROs and provide potential research directions for acupuncture clinical trials.

## 2 Methods

### 2.1 Study design

This cross-sectional study analyzed data from acupuncture clinical trials conducted in mainland China from January 1, 2010, to July 15, 2022, to assess primary and/or secondary outcomes. Data were sourced from ClinicalTrials.gov and the Chinese Clinical Trial Registry. The study only retrieved interventional studies from these two databases (search strategy in Supplementary Method S1). Given this data, the focus of this study was on the continuous use of PRO tools under these conditions. This study followed the Strengthening the Reporting of Observational Studies in Epidemiology (STROBE) reporting guidelines (15).

### 2.2 Data collection strategy

The criteria for inclusion were: (1) topics on acupuncture randomized clinical trials conducted or recruited in mainland China; (2) English and Chinese Language; (3) document types: only include original trials; (4) the design of the trials: outcomes reported PROs; (5) the participants who were over 18 years old met the inclusion criteria. The exclusion criteria included: (1) irrelevant full texts; (2) duplicated registration (retaining ClinicalTrials.gov); (3) outcomes information is incomplete; (4) the design was implemented outside mainland China; (5) the participants who were under 18 years old. The information collected to assess the conditions and characteristics of the trials included (1) basic information, including registration number, registration date, official name, and country; (2) key information, such as outcomes (including PROs), target disease, participant age, and gender; (3) characteristic information, such as the main sponsor, main sponsor’s address, recruitment country, research venue, and the stage of the clinical trial.

### 2.3 Data classification

Eligible trials were categorized into four groups based on the outcomes reported in the study: (1) trials where PROs were listed as primary outcomes, (2) trials where PROs were listed as secondary outcomes, (3) trials that included PROs as both primary and secondary outcomes, and (4) trials that did not mention any PRO tools (the trial registration did not mention the use of PROs).

### 2.4 Statistical analysis

The data from the included trials were independently extracted by two authors (DY and SZ) using a pre-designed data extraction form. The clinical trial phase, research venue, age and gender of the subjects, region of the main sponsor, and research center are shown in Table 1. We classified similar target diseases into the same group according to the International Classification of Diseases-11th Edition (ICD-11) (16).

**Table 1:** Characteristics of all trials and trials including PROs

Characteristics	Total, No. (%)	Trials using PROs
	Trials	
	962	743
Clinical trial phases		
Early stage	281 (29.2%)	221 (29.7%)
1	45 (4.7%)	31 (4.2%)
2	27 (2.8%)	25 (3.4%)
3	17 (1.8%)	14 (1.9%)
4	343 (35.7%)	260 (35.0%)
Other	249 (25.9%)	192 (25.8%)
Study settings		
Hospital	862 (89.6%)	664 (89.4%)
Community	4 (0.4%)	4 (0.5%)
Other	96 (10.0%)	75 (10.1%)
Age		
18-no limit	831 (86.4%)	640 (86.1%)
Over 65	0 (0%)	0 (0%)
Unclear	131 (13.6%)	103 (13.9%)
Gender		
Male	43 (4.4%)	30 (4.0%)
Female	137 (14.2%)	96 (12.9%)
Both	781 (81.1%)	616 (82.9%)
Unclear	1 (0.1%)	1 (0.1%)
Regions, mainland China		
Southwest	122 (12.7%)	96 (12.9%)
Northeast	32 (3.3%)	25 (3.4%)
Northwest	31 (3.2%)	22 (3.0%)
North	254 (26.4%)	199 (26.8%)
East	363 (37.7%)	284 (38.2%)
South	114 (11.9%)	85 (11.4%)
Central	43 (4.5%)	32 (4.3%)
Other	3 (0.3%)	0 (0.0%)
No. of test centers		
Single-center	772 (80.2%)	602 (81.0%)
Multi-center	185 (19.2%)	139 (18.7%)
Unclear	5 (0.5%)	2 (0.3%)
Placebo	144 (15.0%)	113 (15.2%)

Based on our inclusion criteria, our study summarized the PROs used in each trial to calculate the most commonly used measurement tools. We only included items that listed the names of PRO tools in the statistical analysis for a quantitative analysis to understand which assessment tools were used. We used Microsoft Excel 2020 (Microsoft Corporation, Redmond, WA, United States) and python3.9 (Netherlands) to analyze and display the information of PRO.

## 3 Results

### 3.1 Trial characteristics

**Figure 1:**
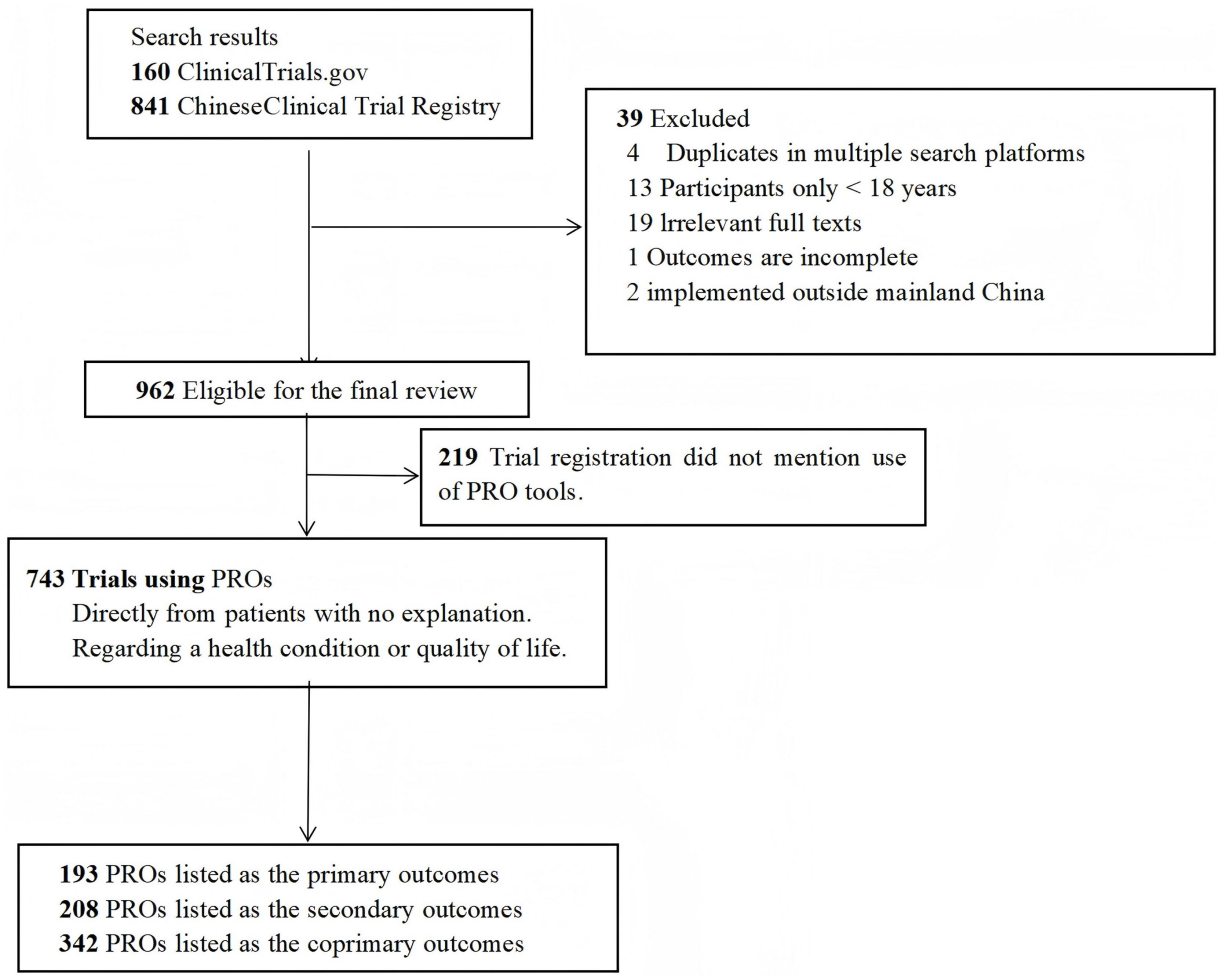
Trial exclusion and classification criteria.

The general characteristics of the included trials are depicted in (Figure 1). We identified a total of 1,001 acupuncture clinical trials conducted in Mainland China, comprising 160 trials from Chictr.org.cn and 841 trials from ClinicalTrials.gov. Our study excluded 39 trials, which included 4 duplicate trials and 13 clinical trials involving participants under the age of 18. A total of 743 trials (77.2%) utilized PRO tools as primary and/or secondary endpoints, while 219 trials (22.8%) did not mention the use of PRO tools.

**Figure 2:**
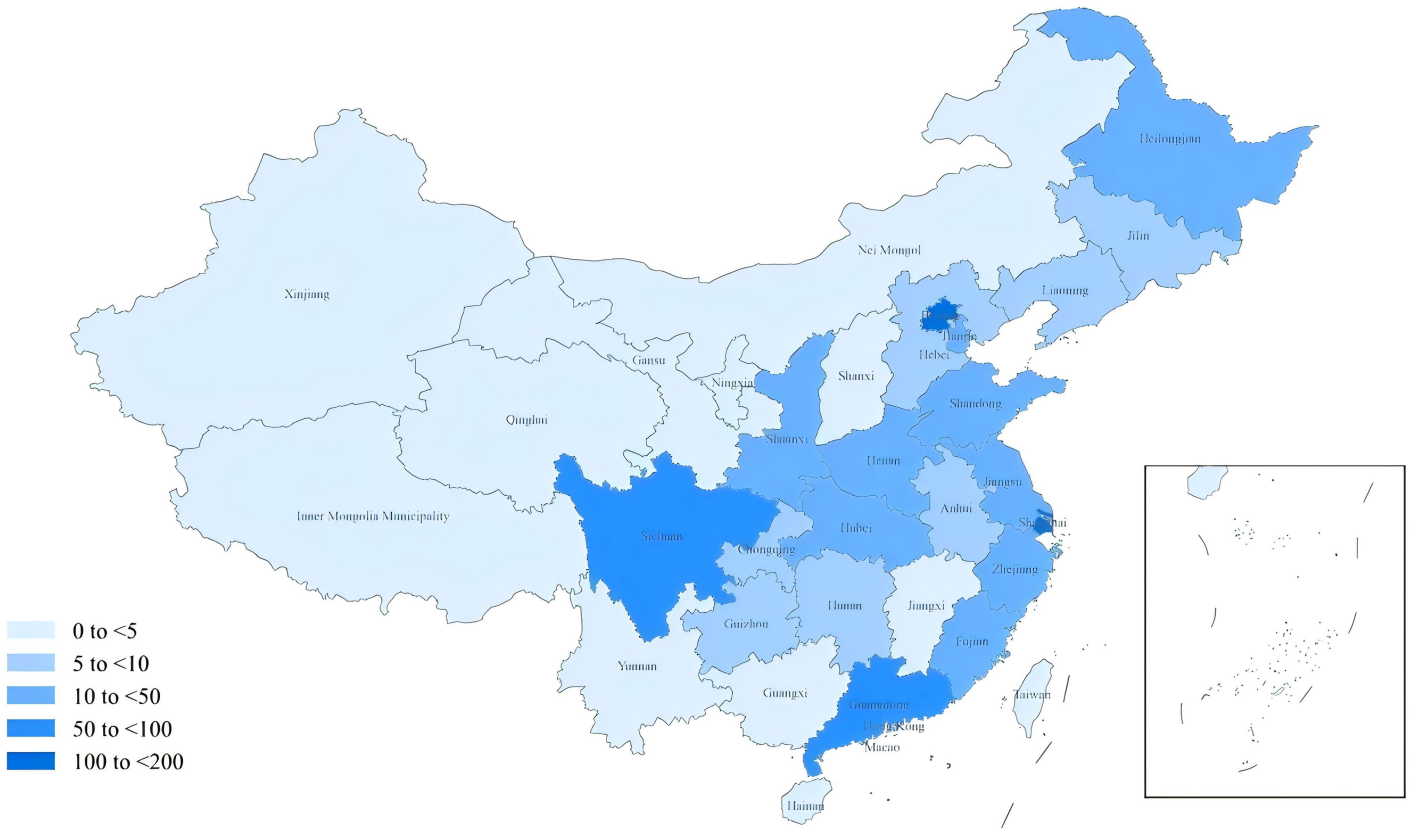
The number of acupuncture trials with Patient-reported outcomes in each province.

Among all clinical trials, phase-4 trials (35.7%) were the most common, followed by early-stage trials (29.2%). Among the 743 trials that reported PROs, phase-4 trials (35.0%) were the most prevalent, and nearly 89.4% of the trials were conducted in hospital settings, and 0.4% were taken placed in the community. The principal investigators of the clinical trial research were predominantly located in the eastern region of mainland China, accounting for 37.7%, followed by the northern region at 26.4% and the southwestern region at 12.7%. Other regions, including the central, northeastern, and northwestern parts of mainland China, constituted 3–5%. When considering only trials that reported PROs, nearly 77.9% (579/743) of the principal investigators were from the eastern, northern, and southwestern regions of mainland China; those from the central, northeastern, and northwestern regions constituted <10.6%. This study also tallied the number of research centers involved in the included acupuncture trials. 772 (80.2%) trials were conducted at a single center, while 19.2% were multi-center trials. Among the acupuncture clinical trials report PROs, 81% were single-center and 18.7% were multi-center. Sham acupuncture was used in the control group of 114 (15.0%) acupuncture trials, with 113 (15.2%) of the acupuncture clinical trials reporting PRO (Table 1). The proportion of principal investigators initiating trials that include PROs varies significantly among different provinces in mainland China. As shown in Figure 2.

### 3.2 Disease status and PROS

**Figure 3:**
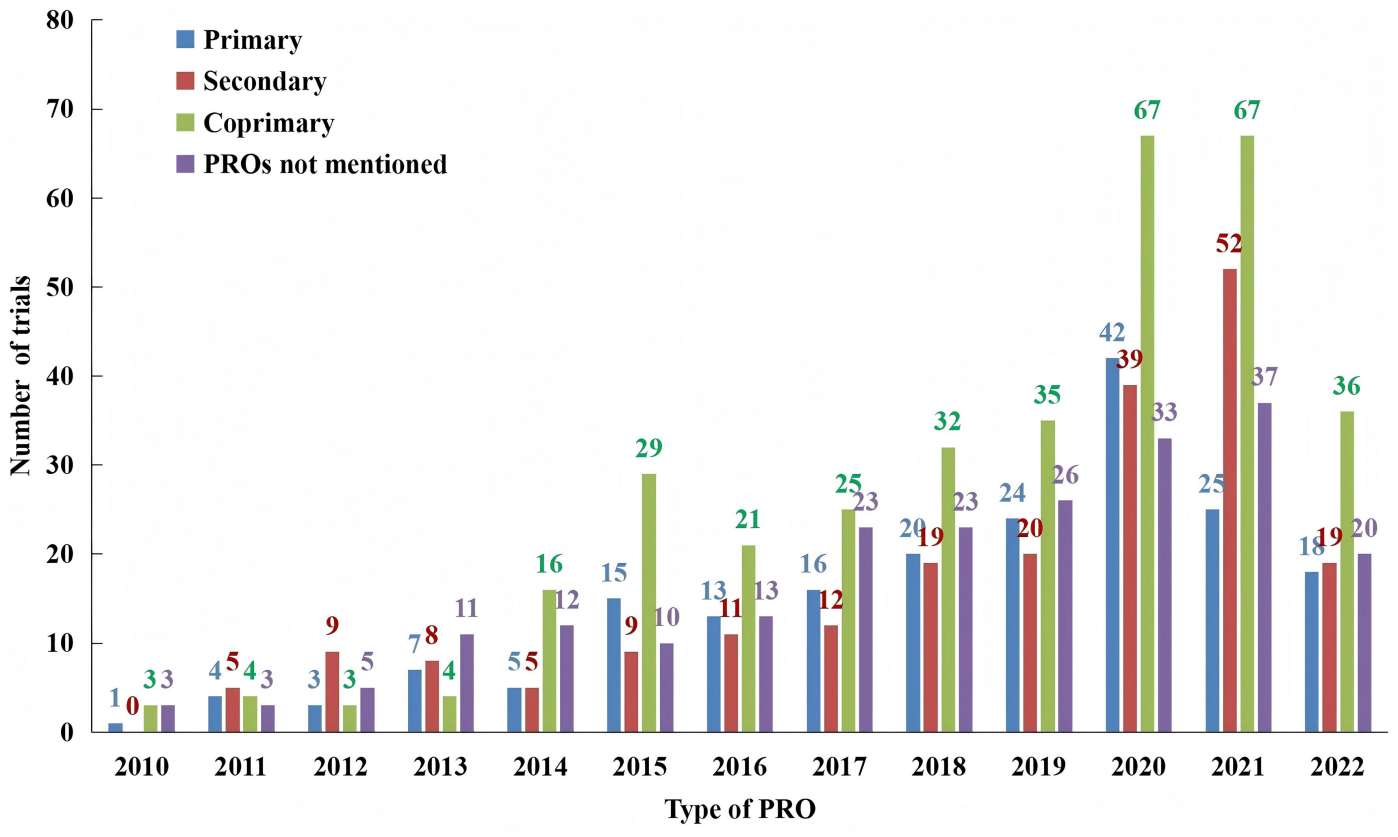
Number of clinical trials analyzed.

Figure 3 illustrates the increase in the number of acupuncture clinical trials registered from 2010 to 2022, and it also showed the proportion of trials that reported PROs as outcome measures within the field of acupuncture. Overall, there has been a significant rise in the use of PROs in acupuncture clinical trials since 2019.

**Figure 4:**
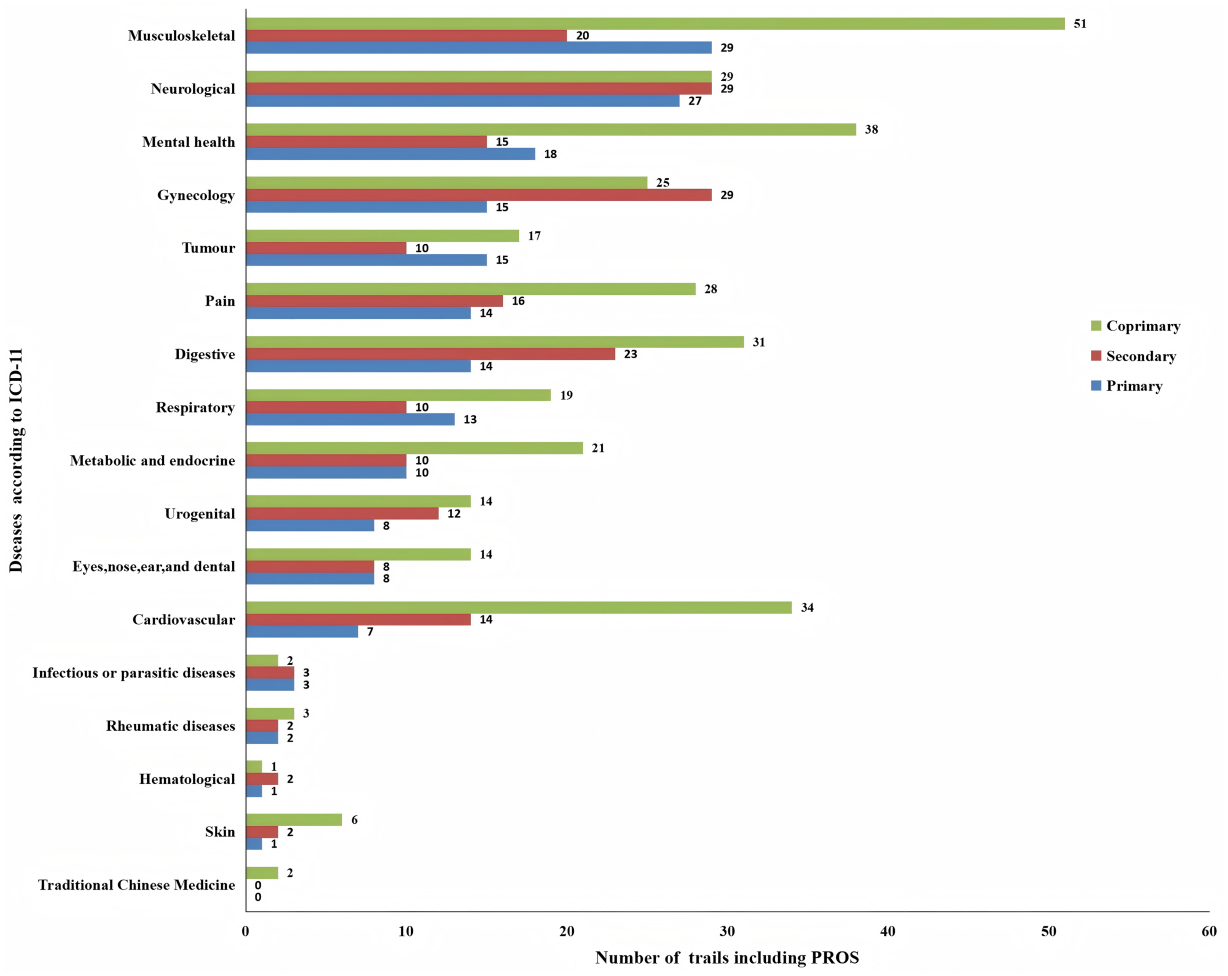
Number of trials with patient-reported outcomes.

Figure 4 illustrates that among the 743 trials utilizing PROs, the top four disease areas applying PRO measurements as outcomes were musculoskeletal disorders (13.3%), neurological disorders (11.3%), mental health conditions (9.6%) and gynecological conditions (9.3%). Based on the type of disease, the most commonly used PRO tools in these trials were identified as the Visual Analogue Scale (VAS), the 36-Item Short Form Survey (SF-36), and the Pittsburgh Sleep Quality Index (PSQI).

Table 2 shows the classification based on the content of the scales used. A total of 664 trials reported symptoms, with 163 of these trials using symptom measures as the primary outcome, 176 as the secondary outcome, and 325 as co-primary outcomes. The trials reported in 196 cases primarily focused on function. Of these, 47 trials used measures of function as the primary outcome, 40 trials as the secondary outcome, and 109 trials as co-primary outcomes. Among the 343 trials related to Health-Related Quality of Life (HRQOL), 47 (24.4%) were based on primary outcomes associated with HRQOL.

**Table 2:** Classification based on the content of the patient-reported outcomes assessment.

PRO instruments conditions					
Trials conditions	Proportion No. (%)	Symptoms	Function	HRQOL	Other
Total no.	743	664	196	343	84
Primary	193	163/193 (84.5%)	47/193 (24.4%)	47/193 (24.4%)	6/193 (3.1%)
Secondary	208	176/208 (84.6%)	40/208 (19.2%)	103/208 (49.5%)	35/208 (16.8%)
Co-primary	342	325/342 (95.0%)	109/342 (31.9%)	193/342 (56.4%)	43/342 (12.6%)

Tables 3–5 displayes the frequency of use of primary and secondary outcome measures based on PROs across different disease categories in acupuncture trials.

**Table 3:** Frequency of use of PROMs as primary outcome in acupuncture clinical trial classification under different conditions

PRO instruments								
Conditions	Proportion No. (%)	No./total no. (%)	Name	No./total no. (%)	Name	No./total no. (%)	Name	No./total no. (%)
Musculoskeletal	114	100	VAS	24/100 (24.0)	PSQI	7/100 (7.0)	HAMD	5/100 (5.0)
Neurological	111	87	PSQI	8/87 (9.2)	HAMA	6/87 (6.9)	VAS	6/87 (6.9)
Gynecology	96	69	VAS	9/69 (13.0)	SF-36	4/69 (5.8)	PSQI	3/69 (4.3)
Mental health	86	71	VAS	10/71 (14.1)	PSQI	8/71 (11.3)	HAMD	4/71 (5.6)
Digestive	85	68	VAS	10/68 (14.7)	WOMAC	3/68 (4.4)	NDS	3/68 (4.4)
Cardiovascular	75	56	PSQI	10/56 (17.9)	VAS	9/56 (16.1)	WOMAC	4/56 (7.1)
Pain	70	58	VAS	15/58 (25.9)	NRS	5/58 (8.6)	SF-36	4/58 (6.9)
Tumour	58	42	VAS	8/42 (19.0)	HAMD	4/42 (9.5)	PSQI	3/42 (7.1)
Respiratory	57	42	VAS	7/42 (16.7)	PSQI	5/42 (11.9)	SAS	3/42 (7.1)
Metabolic and endocrine	56	41	VAS	5/41 (12.2)	TCMSS	4/41 (9.8)	PSQI	3/41 (7.3)
Urogenital	46	34	PSQI	5/34 (14.7)	VAS	4/34 (11.8)	NIH-CPSI	2/34 (5.9)
Eyes, nose, ear, and dental	35	30	VAS	4/30 (13.3)	TNSS	2/30 (6.7)	symptom scores	2/30 (6.7)
Skin	11	9	VAS	2/9 (22.2)	SF-36	1/9 (11.1)	PSQI	1/9 (11.1)
Infectious or parasitic diseases	11	8	VAS	1/8 (12.5)	VAS	1/8 (12.5)	TCMSS	1/8 (12.5)
Rheumatic diseases	9	7	VAS	2/7 (28.6)	NAS	1/7 (14.3)	MPQ	1/7 (14.3)
Hematology	9	4	IBS-SSS	1/4 (25.0)	Short-term memory and delayed recall	1/4 (25.0)	TMT-A	1/4 (25.0)

VAS, visual analog scale; PSQI, Pittsburgh Sleep Quality Index; HAMD, Hamilton Depression Scale; HAMA, Hamilton Anxiety Rating Scale; SF-36, Short-Form 36-item Health Surve; WOMAC, The Western Ontario and McMaster Universities Osteoarthritis IndexThe Western Ontario and McMaster Universities Osteoarthritis Index; NRS, Numeric Rating Scale; SAS, Self-rating Anxiety Scale; TCMSS, TCM symptom score; MPQ, McGill Pain Questionnaire; IBS-SSS, Irritable Bowel Syndrome Symptom Severity; TNSS, Total nasal symptom score; NIH-CPSI, National Institutes of Health Clinical Symptom Score Index of chronic prostatitis.

**Table 4:** Frequency of use of PROMs as secondary primary outcome in acupuncture clinical trial classification under different conditions

PRO instruments								
Conditions	Proportion No. (%)	No./total no. (%)	Name	No./total no. (%)	Name	No./total no. (%)	Name	No./total no. (%)
Musculoskeletal	114	100	SF-36	11/100 (11.0)	SAS	11/100 (11.0)	SDS	10/100 (10.0)
Neurological	111	87	VAS	6/87 (6.9)	SDS	5/87 (5.7)	MMSE	5/87 (5.7)
Gynecology	96	69	SF-36	10/69 (14.5)	VAS	9/69 (13.0)	SAS	8/69 (11.6)
Mental health	86	71	SAS	10/71 (14.1)	SDS	10/71 (14.1)	PSQI	8/71 (11.3)
Digestive	85	68	SDS	12/68 (17.6)	SAS	12/68 (17.6)	SF-36	10/68 (14.7)
Cardiovascular	75	56	SDS	11/56 (19.6)	SAS	9/56 (16.1)	SF-36	8/56 (14.3)
Pain	70	58	VAS	15/58 (25.9)	SF-36	13/58 (22.4)	HAMA	6/58 (10.3)
Tumour	58	42	SDS	4/42 (9.5)	PSQI	3/42 (7.1)	SAS	3/42 (7.1)
Respiratory	57	42	VAS	7/42 (16.7)	SF-36	6/42 (14.3)	TCMSS	5/42 (11.9)
Metabolic and endocrine	56	41	SF-36	5/41 (12.2)	SAS	4/41 (9.8)	VAS	4/41 (9.8)
Urogenital	46	34	ICIQ-SF	5/34 (14.7)	SDS	5/34 (14.7)	SAS	5/34 (14.7)
Eyes, nose, ear, and dental	35	30	SAS	4/30 (13.3)	VAS	4/30 (13.3)	SDS	3/30 (10.0)
Skin	11	9	MOCA	2/9 (22.2)	quality of life	1/9 (11.1)	MMSE	1/9 (11.1)
Infectious or parasitic diseases	11	8	MOCA	1/8 (12.5)	FAQ	2/8 (25.0)	GCS	1/8 (12.5)
Rheumatic diseases	9	7	SF-36	2/7 (28.6)	NAS	1/7 (14.3)	HAD	1/7 (14.3)
Hematology	9	4	Appetite	1/4 (25.0)	Defecation	1/4 (25.0)	PSQI	1/4 (25.0)

SF-36, Short-Form 36-item Health Surve; SAS, Self-rating Anxiety Scale; SDS, Self-rating Depression Scale; VAS, visual analog scale; MMSE, Mini-mental State Examination; PSQI, Pittsburgh Sleep Quality Index; HAMA, Hamilton Anxiety Rating Scale; TCMSS, TCM symptom score; ICIQ-SF, International Consultation on Incontinence Questionnaire–Short Form; MOCA, Montreal cognitive assessment; MMSE, Mini-mental State Examination.

**Table 5:** Frequency of use of PROMs as co-primary primary outcome in acupuncture clinical trial classification under different conditions

PRO instruments								
Conditions	Proportion No. (%)	No./total no. (%)	Name	No./total no. (%)	Name	No./total no. (%)	Name	No./total no. (%)
Musculoskeletal	114	100	VAS	30/100 (30.0)	PSQI	17/100 (17.0)	SF-36	15/100 (15.0)
Neurological	111	87	PSQI	13/87 (14.9)	VAS	12/87 (13.8)	MMSE	11/87 (12.6)
Gynecology	96	69	VAS	18/69 (26.1)	SF-36	14/69 (20.3)	SAS	10/69 (14.5)
Mental health	86	71	VAS	16/71 (22.5)	SDS	14/71 (19.7)	PSQI	14/71 (19.7)
Digestive	85	68	VAS	17/68 (25.0)	SAS	13/68 (19.1)	SDS	13/68 (19.1)
Cardiovascular	75	56	PSQI	14/56 (25.0)	VAS	12/56 (21.4)	SDS	12/56 (21.4)
Pain	70	58	VAS	26/58 (44.8)	SF-36	17/58 (29.3)	HAMA	6/58 (10.3)
Tumour	58	42	VAS	10/42 (23.8)	PSQI	6/42 (14.3)	SDS	5/42 (11.9)
Respiratory	57	42	VAS	13/42 (31.0)	SF-36	7/42 (16.7)	PSQI	6/42 (14.3)
Metabolic and endocrine	56	41	VAS	8/41 (19.5)	SF-36	6/41 (14.6)	PSQI	6/41 (14.6)
Urogenital	46	34	PSQI	6/34 (17.6)	ICIQ-SF	5/34 (14.7)	VAS	5/34 (14.7)
Eyes, nose, ear, and dental	35	30	VAS	8/30 (26.7)	SAS	4/30 (13.3)	RQLQ	4/30 (13.3)
Skin	11	9	VAS	3/9 (33.3)	NRS	2/9 (22.2)	MOCA	2/9 (22.2)
Infectious or parasitic diseases	11	8	MOCA	2/8 (25.0)	GCS	1/8 (12.5)	BASDAI	1/8 (12.5)
Rheumatic diseases	9	7	VAS	3/7 (42.9)	SF-36	2/7 (28.6)	NAS	1/7 (14.3)
Hematology	9	4	Appetite	1/4 (25.0)	Defecation	1/4 (25.0)	PSQI	1/4 (25.0)

VAS, visual analog scale; PSQI, Pittsburgh Sleep Quality Index; SF-36, Short-Form 36-item HealthSurvey; MMSE, Mini-mental State Examination; SAS, Self-rating Anxiety Scale; SDS, Self-rating Depression Scale; HAMA, Hamilton Anxiety Rating Scale; ICIQ-SF, International Consultation on Incontinence Questionnaire–Short Form; RQLQ, Rhinoconjunctivitis Quality of Life Questionnaire; NRS, numeric rating scale; MOCA, Montreal Cognitive Assessment; GCS; BASDAI, Bath Ankylosing Spondylitis Metrology Index; NAS, numeric rating scale.

Table 3 illustrates that among the 193 trials in which PROs were listed as primary outcomes, musculoskeletal disorders were the most prevalent, accounting for 29 cases (15.0%), followed by neurological disorders (27 cases, 13.9%), and mental health conditions (18 cases, 9.3%). Subsequently, oncological conditions and gynecological diseases each accounted for 15 cases (7.8%), while digestive system diseases and pain conditions each represented 14 cases (7.2%). Lastly, respiratory diseases were associated with 13 cases (6.7%). The least common conditions were infectious or parasitic diseases with 3 cases (1.6%), dermatological conditions with 1 case (0.5%), and rheumatic diseases with 2 cases (1.0%).

Among the 743 trials that included PROs, the Visual Analogue Scale (VAS) (24.0%), Pittsburgh Sleep Quality Index (PSQI) (17.9%), 36-Item Short Form Survey (SF-36) (14.5%), and Self-Rating Anxiety Scale (SAS) (12.5%) were the four most commonly used measurement methods. In terms of primary outcomes, the VAS was widely utilized across various disease categories, particularly in the category of pain where it was used most frequently (24.0%). The PSQI was frequently used in the cardiovascular, oncological, respiratory, and urogenital categories. The HAMD was used more often in the categories of mental health and oncology. Within the “Mental Health” category for primary outcomes, the VAS (14.1%) and PSQI (11.3%) were used with higher frequency.

In terms of secondary outcomes, the SF-36, a widely used measure of health-related quality of life, was frequently utilized across various disease classifications, particularly in musculoskeletal (11.0%), gynecology and obstetrics (14.5%), digestive (14.7%), cardiovascular and cerebrovascular (14.3%), and metabolic and endocrine diseases (12.2%). The SAS was commonly used in mental health (14.1%), digestive system diseases (17.6%), cardiovascular and cerebrovascular conditions (16.1%), urogenital disorders (14.7%), and diseases of the ear, nose, and throat (13.3%). The SDS was also frequently used in the areas of digestive diseases (17.6%), cardiovascular and cerebrovascular diseases (19.6%), and oncology (9.5%).

## 4 Discussion

### 4.1 Summary of findings

This cross-sectional study analyzed the application and characteristics of PRO tools in acupuncture randomized clinical trials conducted in mainland China from 2010 to 2022. In our study, there were significant geographic differences in the use of PROs. Beijing, Shanghai, Sichuan Province, and Guangdong province conducted acupuncture clinical trials using PROs more frequently, and only Heilongjiang province in the northeast region conducted acupuncture clinical trials using PROs in a higher number of trials, and even fewer trials were conducted in the western region. This may be related to the corresponding geographic location’s rapid economic development, advanced medical care, diversity in education and cultural development, and the residents’ pursuit of a healthy quality of life and higher acceptance of PROs (17).

Our findings indicated that only 20% of the trials employed PRO tools to assess patients’ subjective experiences as primary outcomes. Clinical trials must prioritize patients’ interests highly, and it is very important to value patients’ opinions and perspectives. Acupuncture clinical researchers need to recognize the significant role of incorporating PROs into the evaluation of treatment efficacy in acupuncture clinical trials. This can not only better highlight the distinctive advantages of acupuncture but also enhance the comprehensive accuracy of efficacy assessment, as well as contribute to the improvement and optimization of acupuncture treatment regimens (18).

Our research has shown that PRO has been reported primarily in acupuncture clinical trials for musculoskeletal disorders, neurological disorders, and mental health conditions. Acupuncture has been shown to treat musculoskeletal disorders, neurological disorders, and mental health conditions with superior efficacy in reducing pain, relieving anxiety, and regulating moods (19, 20).

The evaluation tools are mainly the VAS scale, SF-36 scale, and PSQI scale. The effect of acupuncture treatment depends greatly on the subjective experience of the patient, such as pain and relaxation. The PRO tools can assess the patient’s immediate feelings after acupuncture treatment from the patient’s point of view (21). The effects of acupuncture treatment may persist even after the treatment has ended and change over time. The PRO tools can be used to track the health status of patients at different time points and to assess the long-term effects of acupuncture to verify the difference in efficacy between acupuncture and sham acupuncture. The PRO tools can be used to assess the effects of acupuncture from the patient’s point of view (22).

The use of the VAS scale is highlighted among the assessment tools for primary versus secondary outcomes. The VAS is a simple and rapid patient self-report scoring tool that assesses pain intensity more visually, is easily understood by patients, and is simple to administer (23). In our study, the SF-36 was found to be the main PRO tool used to measure quality of life. There is a consensus among TCM practitioners that existing quality-of-life instruments may not be sensitive enough to detect health changes that are considered important in acupuncture treatments (24).

Clinical trial outcomes are categorized into four categories: symptoms, function, health-related quality of life (HRQOL), and others. Outcomes related to clinical manifestations such as pain, and insomnia are categorized as symptoms. The category “function” includes concepts such as physical functioning, activity limitation, and emotional function. “HRQOL” refers to high-level concepts such as quality of life, HRQOL, and perceived wellbeing. “Other” contains concepts such as satisfaction, and preferences (25).

Our research indicated that in acupuncture clinical trials, the majority of PRO tools are used to assess symptoms and quality of life. Acupuncture not only focuses on the improvement of symptoms but also emphasizes the enhancement of overall health and quality of life. PRO tools are capable of directly collecting information from patients regarding their health status and quality of life, which aligns well with the holistic treatment objectives of acupuncture.

The study revealed that only a small number of PRO tools are utilized to evaluate patient satisfaction with the treatment. Patient preferences and satisfaction with acupuncture treatment, as well as their expectations for therapeutic outcomes, are integral components of assessing the effectiveness of acupuncture. These factors may aid in the development of more precise placebo controls and blinding methods, and in elucidating the contribution of placebo effects to the therapeutic effects of acupuncture (26). It is recommended that researchers place importance on investigating patient satisfaction with acupuncture treatment plans within acupuncture research.

In clinical trials of acupuncture in which sham acupuncture served as a control group, 78% reported the use of PROs. Sham acupuncture is closely associated with PROs, because PROs provide direct evidence for assessing therapeutic efficacy and help to scientifically compare the effects of real acupuncture with sham acupuncture.

### 4.2 Strengths and limitations

This study conducts an in-depth investigation into the application status of PROs in acupuncture randomized clinical trials in mainland China from 2010 to 2022, providing the first systematic investigation of the characteristics of PROs utilization in acupuncture clinical trials. However, there are several limitations to our research. Firstly, we exclude trials involving pediatric populations due to the potential inability of children to accurately articulate their genuine experiences, thereby avoiding potential bias in the results. Secondly, our research identifies that some trials’ registration information is not updated promptly, remaining in a recruitment status, which may lead to biases in the statistical analysis.

### 4.3 Comparison with previous research

No systematic studies that elucidate the application characteristics of PROs in acupuncture clinical trials have been identified. Previous literature has examined the use of PROs in modern medical fields such as oncology, endocrinology, and cardiovascular diseases as well as in the field of complementary and alternative medicine including TCM, homeopathic, mindfulness and so on (27, 28). However, acupuncture should be individually and systematically excavated due to its complex holistic diagnostic and therapeutic characteristics.

### 4.4 Implications for future research

In this cross-sectional study, the use of PROs has increased in acupuncture clinical trials conducted in mainland China. However, we observe geographic disparities in the application of PROs in acupuncture clinical trials, and there is currently a lack of standardized PRO tools tailored to the specific characteristics of acupuncture. We recommend that acupuncture clinical researchers place importance on the use of PRO tools in clinical trials. Furthermore, considering the applicability across different cultures (29), there is a need to further integrate regional acupuncture-specific content and to research and develop PRO scales that conform to the inherent patterns and characteristics of acupuncture such as patient responses to “de qi” and individualized pattern differentiation. Establishing a clinical evidence-based evaluation system that aligns with the features of acupuncture can enhance the quality and reliability of the research outcomes.

## Data Availability

The original contributions presented in the study are included in the article/Supplementary material, further inquiries can be directed to the corresponding author.
