# Supplementary material for: Patient-reported outcome tools of acupuncture clinical trials in mainland China: a cross-sectional study
Source: Front Neurol. 2025 Jun 6;16:1520759. doi: 10.3389/fneur.2025.1520759 (PMC12180298; doi:10.3389/fneur.2025.1520759)
Supplement: Supplementary file 1 [file Data_Sheet_1.pdf]

Supplement

Methods. Search strategy

1. Search strategy for www.chictr.org.cn
"Study type" = Interventional Study
"Study phase" = 1, 2, 3, 4, 1-2, 2-3, 0, 4, N/A,
"First Posted" = "01/01/2010" To "15/07/2022"
"sex" = Male, Female, Both
"Country" = China
"Intervention/treatment"= acupuncture OR electroacupuncture OR Intradermal acupuncture OR warm acupunctrue OR ear acupuncture OR manual acupuncture OR abdominal acupuncture OR ture acupuncture OR sham acupuncture

2. Search strategy for ClinicalTrials.gov
"Study type" = Interventional Studies (Clinical Trials)
"Phase" = Early phase 1, Phase 2, Phase 3, Phase 4, Not Applicable
"First Posted" = "01/01/2010" To "31/12/2020"
"Sex" = Studies with Male Participants, Studies with Female Participants, All
"Country" = China
"Intervention/treatment"= acupuncture OR electroacupuncture OR Intradermal acupuncture OR warm acupunctrue OR ear acupuncture OR manual acupuncture OR abdominal acupuncture OR ture acupuncture OR sham acupuncture
